# Long-term immunogenicity and safety after a single dose of the quadrivalent meningococcal serogroups A, C, W, and Y tetanus toxoid conjugate vaccine in adolescents and adults: 5-year follow-up of an open, randomized trial

**DOI:** 10.1186/s12879-015-1138-y

**Published:** 2015-10-06

**Authors:** Charissa Fay Corazon Borja-Tabora, Cecilia Montalban, Ziad A. Memish, Dominique Boutriau, Devayani Kolhe, Jacqueline M. Miller, Marie Van der Wielen

**Affiliations:** Research Institute for Tropical Medicine, Corporate Ave, Muntinlupa City, 1781 Metro Manila Philippines; Philippine General Hospital Manila, Taft Avenue, Ermita, Manila, 1000 Metro Manila Philippines; College of Medicine, Alfaisal University, Al Zahrawi Street, Al Maather, Al Takhassusi Rd, Riyadh, 11533 Saudi Arabia; GSK Vaccines, Avenue Fleming 20, B- 1300 Wavre, Belgium; GSK Pharmaceuticals Limited, Embassy Links, #5, SRT Road, Bangalore, 560052 India; GSK Vaccines, 2301 Renaissance Blvd, King of Prussia, PA 19406 USA

**Keywords:** Quadrivalent meningococcal conjugate vaccine, Bactericidal antibody, Antibody persistence, Safety

## Abstract

**Background:**

Long-term protection against meningococcal disease is associated with persistence of post-vaccination antibodies at protective levels. We evaluated the bactericidal antibody persistence and safety of the quadrivalent meningococcal serogroups A, C, W and Y tetanus-toxoid conjugate vaccine (MenACWY-TT) and the meningococcal polysaccharide serogroups A, C, W, and Y vaccine (MenACWY-PS) up to 5 years post-vaccination.

**Methods:**

This phase IIb, open, randomized, controlled study conducted in the Philippines and Saudi Arabia consisted of a vaccination phase and a long-term persistence phase. Healthy adolescents and adults aged 11–55 years were randomized (3:1) to receive a single dose of MenACWY-TT (ACWY-TT group) or MenACWY-PS (Men-PS group). Primary and persistence results up to 3 years post-vaccination have been previously reported. Antibody responses against meningococcal serogroups A, C, W, and Y were assessed by a serum bactericidal antibody assay using rabbit complement (rSBA, cut-off titers 1:8 and 1:128) at Year 4 and Year 5 post-vaccination. Vaccine-related serious adverse events (SAEs) and cases of meningococcal disease were assessed up to Year 5.

**Results:**

Of the 500 vaccinated participants, 404 returned for the Year 5 study visit (Total Cohort Year 5). For the Total Cohort Year 5, 71.6–90.0 and 64.9–86.3 % of MenACWY-TT recipients had rSBA titers ≥1:8 and ≥1:128, respectively, compared to 24.8–74.3 and 21.0–68.6 % of MenACWY-PS recipients. The rSBA geometric mean titers (GMTs) remained above the pre-vaccination levels in both treatment groups. Exploratory analyses suggested that both rSBA GMTs as well as the percentages of participants with rSBA titers above the cut-offs were higher in the ACWY-TT than in the Men-PS group for serogroups A, W and Y, with no apparent difference for MenC. No SAEs related to vaccination or cases of meningococcal disease were reported up to Year 5.

**Conclusion:**

These results suggest that a single dose of MenACWY-TT could protect at least 72 % of vaccinated adolescents and adults against meningococcal disease at least 5 years post-vaccination.

**Trial registration:**

ClinicalTrials.gov NCT00356369

**Electronic supplementary material:**

The online version of this article (doi:10.1186/s12879-015-1138-y) contains supplementary material, which is available to authorized users.

## Background

*Neisseria meningitidis* serogroups A, B, C, W, Y and X account for the majority of invasive meningococcal infections, which are associated with high morbidity and mortality rates [1–3]. There are both geographical and temporal variations in the distribution of these serogroups; these variations are potentially influenced by international travel patterns [1–4]. The risk for infection is higher during disease outbreaks and for people living in or traveling to areas with a higher incidence of endemic disease (e.g. the meningitis belt of Africa) [5–7]. In Asia and the Middle East, serogroups A, C and W are predominant [2]. In the Philippines, serogroup A was responsible for an outbreak of meningococcal disease in 2004–2005 [8]. In Saudi Arabia, outbreaks of meningococcal disease associated with mass gatherings during the Hajj or Umrah pilgrimages facilitating person-to-person transmission have repeatedly occurred [9–11]. Serogroup A was responsible for an outbreak following the Hajj in 1987 [12], while serogroup W was predominant during outbreaks in 2000 and 2001 [4, 9, 12].

To reduce the burden of disease, meningococcal polysaccharide (MenPS) vaccines were successfully introduced more than 3 decades ago [13]. However, MenPS vaccines have some limitations, including poor immunogenicity in infants and toddlers, a lack of capacity to induce long-term protection or immune memory, a negligible impact on nasopharyngeal carriage, failure to confer herd immunity, and observed hyporesponsiveness after repeated doses [14, 15]. To overcome these drawbacks, capsular polysaccharides can be coupled to carrier proteins as demonstrated by monovalent meningococcal serogroup C conjugate vaccines [16–20]. Moreover, vaccination against multiple serogroups may be the best strategy to protect individuals against a broader range of meningococcal diseases in a single injection.

Currently, three quadrivalent meningococcal conjugate vaccines (MenACWY) are available [15]: a tetanus toxoid (TT) conjugate vaccine (MenACWY-TT; Nimenrix™, GSK Vaccines, Rixensart, Belgium); a diphtheria toxoid (DT) conjugate vaccine (MenACWY-DT; *Menactra*^TM^, Sanofi Pasteur Inc., Swiftwater, Pennsylvania); and a nontoxic mutant variant of *Corynebacterium diphtheriae* toxin (CRM_197_) conjugate vaccine (MenACWY-CRM; *Menveo*^TM^, GSK Vaccines, Rixensart, Belgium). Previous clinical studies have shown that MenACWY-TT is immunogenic and well-tolerated in toddlers, children, adolescents, and adults, and that functional antibodies induced by a single vaccine dose persist up to 3 years after vaccination [21–35].

In both the Philippines and Saudi Arabia, meningococcal vaccination is recommended for individuals who are at increased risk for meningococcal infection. After the 2000 and 2001 disease outbreaks, Saudi Arabia changed the vaccination recommendation from a bivalent meningococcal serogroup A and C polysaccharide vaccine to the quadrivalent (A, C, W and Y) polysaccharide vaccine as a Hajj visa requirement [4, 36, 37]. No further Hajj or Umrah related outbreaks have occurred since then, and the incidence of invasive meningococcal disease was reduced in the region [12, 36, 38]. Since January 2013, routine immunization of children older than 2 years of age and vaccination of Hajj pilgrims with quadrivalent MenPS or conjugate vaccines is recommended in Saudi Arabia [39]. Hajj pilgrims are required to have the meningococcal vaccine ≤3 years and ≥10 days before arriving in Saudi Arabia. Since individuals may participate in multiple pilgrimages, the conjugate vaccine is preferred to the MenPS vaccine due to concerns of hyporesponsiveness with repeated doses [40]. In the Philippines, meningococcal vaccines are not included in the national routine immunization schedule, but vaccination with quadrivalent polysaccharide (single dose) or conjugate vaccines (single dose in adults, 2 doses in children aged ≥9 months given at 2-month intervals; boosters at 5-year intervals) is recommended by both pediatric and adult infectious disease societies for individuals who are at increased risk for meningococcal infection [41].

The introduction of quadrivalent meningococcal conjugate vaccines has led to the reduction of the number of cases of invasive disease. However, several studies have shown that serum antibody levels elicited by conjugate vaccines wane over time, especially at younger ages [42–44]. Previous clinical data have shown that the ability of meningococcal serogroup C conjugate vaccines to induce immunologic memory does not necessarily confer protection, because the onset of invasive meningococcal disease may occur before sufficient antibodies are produced [16, 45]. Therefore, the maintenance of persisting antibodies may be a more reliable predictor than immune memory for long-term protection against meningococcal disease [45].

Results of a randomized study enrolling 500 participants showed MenACWY-TT to be non-inferior compared to a licensed MenACWY plain polysaccharide vaccine (MenACWY-PS; *Mencevax*™, GSK Vaccines, Belgium) in terms of rSBA vaccine response to the four meningococcal serogroups one month after vaccination. The primary outcome of the study was thus met and results showing that a single dose of MenACWY-TT induced bactericidal antibodies which persisted in a majority of participants, and was well-tolerated up to 3 years post-vaccination were reported in a previous publication [30]. The secondary outcomes described in this publication include the comparison of the long-term persistence of immunogenicity of the two vaccines, and the description of serious adverse events (SAEs) related to vaccination and of any event related to the lack of vaccine efficacy up to Year 5 after vaccination.

## Methods

### Study design and participants

This phase IIb, open, randomized, controlled study was conducted between December 2006 and February 2013 in two centers in the Philippines (Research Institute for Tropical Medicine in Muntilupa City and Philippine General Hospital in Manila) and one center in Saudi Arabia (King Abdulaziz Medical City in Riyadh). The study consisted of two phases: the vaccination phase (www.clinicaltrials.gov, NCT00356369) and the long-term persistence phase (up to 5 years after vaccination).

Study participants were healthy adolescents and adults aged 11–55 years at the time of vaccination, who had completed routine childhood vaccination to the best of their or their parents’/guardians’ knowledge. At each yearly visit, participants were excluded from the long-term persistence phase of this study if they had received a meningococcal vaccine not permitted in the protocol or if they had a history of meningococcal disease. Participants with a suboptimal response to all four serogroups at a post-vaccination timepoint received an additional vaccine dose and were excluded from the study. Participants with a suboptimal response to one of the four serogroups were not excluded from the study, unless the study investigator considered that the epidemiological risks warranted the administration of an additional vaccine dose.

In the vaccination phase, participants were randomized (3:1) to two groups receiving either a single dose of MenACWY-TT (ACWY-TT group) or MenACWY-PS (Men-PS group); the randomization procedure, accounting for study center and age of the participant (11–17 and 18–55 years of age), as well as the composition and administration of these vaccines, has been previously described [30]. Here, we present results at Years 4 and 5 after vaccination, evaluated between January 2011 and February 2013.

The study was conducted in accordance with the Good Clinical Practice Guidelines and the Declaration of Helsinki, and the protocol and associated documents were reviewed and approved by the following local ethics committees: the Research Institute for Tropical Medicine (RITM) Institutional Review Board, Muntinlupa City, Philippines; University of the Philippines Manila Research Ethics Board, Manila, Philippines; and the National Guard Health Affair Institutional Review Board (IRB), King Abdulaziz Medical City, Riyadh, Kingdom of Saudi Arabia. Written informed consent was obtained before enrolment from the participants aged at least 16 years in Saudi Arabia and 18 years in the Philippines. Younger participants signed a written informed assent and a written informed consent was obtained from their parents/guardians. In addition, consent was obtained if participants reached 18 years of age during the study. A summary of the protocol is available at http://www.gsk-clinicalstudyregister.com (GSK study ID 107386).

### Immunogenicity assessments

Blood samples were collected from all participants at Years 4 and 5. Functional antibody responses against meningococcal serogroups A, C, W and Y were assessed by a serum bactericidal activity assay with rabbit complement as exogenous complement source (rSBA, cut-off titer 1:8) [46]. rSBA titers ≥1:8 correlate with short-term protection for serogroup C [47], and this threshold was extended to the other serogroups. The percentages of participants with rSBA titers ≥1:128, a more conservative correlate of protection, were also evaluated [48]. The assay for Years 4 and 5 was performed at the Public Health England (PHE) laboratory. This prevented a direct comparison with the results from earlier timepoints, which were assessed at the GSK Vaccines laboratory, for the entire study cohort. For a subset of participants, samples from previous timepoints were retested at the PHE laboratory for direct comparison.

### Safety assessments

During the long-term persistence phase, only SAEs that were considered by the investigator to have a causal relationship to the vaccination were collected retrospectively up to Year 5. The occurrence of meningococcal disease, as an event related to the lack of vaccine efficacy, was also described. Any pregnancy complication or elective termination of a pregnancy for medical reasons or spontaneous abortion in a study participant was recorded.

### Statistical analyses

Two cohorts were defined for the purpose of the antibody persistence analysis. The Total Cohort Year 5 included all vaccinated participants who attended the Year 5 follow-up visit and for whom data concerning persistence endpoint measures were available. The according-to-protocol (ATP) cohort for persistence Year 5 included all evaluable participants who received the vaccine during the vaccination phase, had not received a previous dose of meningococcal serogroup A, C, W or Y vaccines not planned in the protocol, were compliant with protocol-defined windows for serum sampling (254 to 266 weeks post-vaccination), and had available assay results for at least one tested antigen at the Year 5 timepoint.

The percentages of participants with antibody titers above the proposed cut-offs and the geometric mean antibody titers (GMTs) for the tested antigens were calculated with 95 % confidence intervals (CIs). The exact 95 % CIs for proportion within a group were calculated based on the method described by Clopper et al. [49]. The standardized asymptotic 95 % CIs for the group difference in proportions were calculated based on the method 6 described by Newcombe [50]. Antibody titers below the cut-off of the assay (1:8) were given an arbitrary value of half the cut-off for the purpose of GMT calculation.

All comparative analyses between groups were exploratory. Potential differences between groups were identified if the standardized asymptotic 95 % CI for (1) the difference in percentage of participants with titers above the proposed cut-offs between the two groups did not contain the value ‘0’ or (2) the GMT ratio between the two groups did not contain the value ‘1’. Since no adjustment for multiplicity of these comparisons was performed, the results should be interpreted with caution.

The analyses were performed using Statistical Analysis System version 9.22 for Windows (SAS Institute Inc., Cary, NC, United States).

## Results

### Demographics

**Fig. 1:**
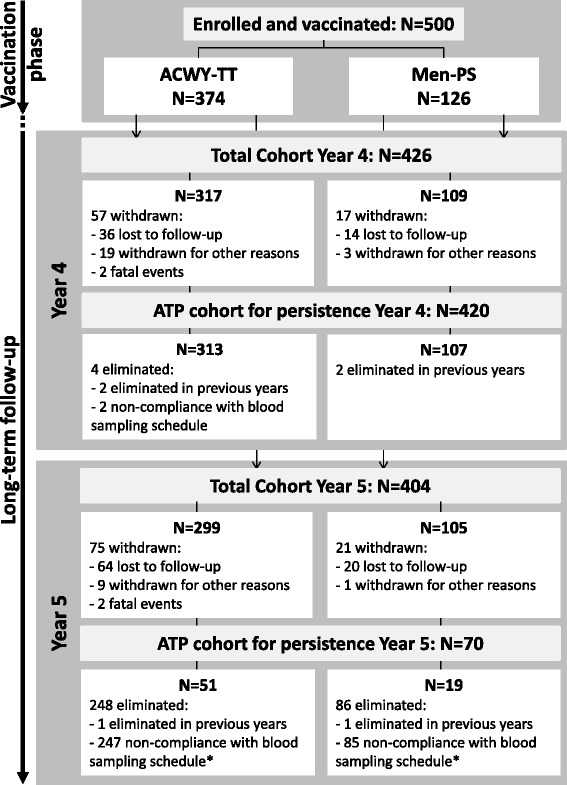
Flow of participants. ACWY-TT = group of participants who received one dose of MenACWY-TT at Month 0; Men-PS = group of participants who received one dose of MenACWY-PS at Month 0; *N* = number of participants; ATP = according to protocol. *Due to a delay in the annual approval from the FDA of the Philippines for the Year 5 persistence timepoint, all participants enrolled in the Philippines attended the Year 5 visit outside protocol-defined windows between the primary vaccination and the Year 5 blood sampling and hence were eliminated from the ATP analysis at Year 5 because of non-compliance with the blood sampling schedule

Of the 500 participants who were vaccinated in the primary phase of the study, 426 returned for the Year 4 study visit and 404 for the Year 5 visit. The ATP cohort for persistence Year 4 included 420 participants (313 in the ACWY-TT group and 107 in Men-PS group) and the Year 5 ATP cohort included 70 participants (51 in the ACWY-TT group and 19 in Men-PS group) (Fig. 1). This low number of participants included in the ATP cohort at Year 5 was due to a delay in the annual approval from the Food and Drug Administration (FDA) of the Philippines for the Year 5 persistence timepoint, which caused all 333 participants enrolled in the Philippines to attend the Year 5 visit outside the protocol-defined windows for serum sampling (254 to 266 weeks post-vaccination); they were therefore eliminated from the ATP analysis. In the Total Cohort Year 5, the mean interval between vaccination and the Year 5 serum sampling was ≈ 288 weeks (range 258 to 301 weeks) post-vaccination.

At the time of vaccination, 301 participants were 11–17 years old and 199 participants were 18–55 years old. The age and gender distribution of the participants in the Total Cohort Year 5 were similar in both groups, and were consistent with those of the participants enrolled in the vaccination phase of the study (Table 1). The population in the Total Cohort Year 5 was predominantly of South East Asian heritage (82.4 %).

**Table 1:** Demographic characteristics of the study participants (Total Cohort Year 5)

Characteristics	Parameter or category	ACWY-TT	Men-PS
		(*N* = 299)	(*N* = 105)
Age at Year 5 (years)	mean age (SD)	23.3 (7.7)	23.6 (8.1)
Age stratum (years)	11–17, *n* (%)	208 (69.6)	76 (72.4)
	18–55, *n* (%)	91 (30.4)	29 (27.6)
Gender	Female, *n* (%)	136 (45.5)	52 (49.5)
Race	Asian - South East Asian heritage, *n* (%)	247 (82.6)	86 (81.9)
	White - Arabic/North African heritage, *n* (%)	52 (17.4)	19 (18.1)

ACWY-TT = group of participants who received one dose of MenACWY-TT at Month 0

Men-PS = group of participants who received one dose of MenACWY-PS at Month 0

*N* = total number of participants

SD = standard deviation

*n* (%) = number (percentage) of participants in a given category

### Persistence of bactericidal antibodies

The primary analysis of immunogenicity was based on the ATP cohort for persistence Year 5 (Additional file 1: Table S1). Because none of the participants from the Philippines were included in this cohort due to non-compliance with the blood sampling schedule, the Total Cohort Year 5 was considered to be more representative for the study population, and the results based on this cohort are presented in this manuscript.

In the Total Cohort Year 5, the percentages of participants with seroprotective rSBA titers, as measured by the PHE assay at Year 5, ranged from 71.6 to 90.0 % in the ACWY-TT group, and from 24.8 to 74.3 % in the Men-PS group (Table 2). The percentages of participants with rSBA titers ≥1:128 ranged from 64.9 to 86.3 % in the ACWY-TT group and from 21.0 to 68.6 % in the Men-PS group. At Year 4, 5 participants (3 from the ACWY-TT group, 2 from the Men-PS group) and at Year 5, 3 participants (1 from ACWY-TT group, 2 from the Men-PS group) had a suboptimal response to all 4 serogroups. These participants received an additional dose of a licensed meningococcal vaccine and were excluded from the long-term follow-up of that particular year and the following years.

**Table 2:** Percentage of participants with rSBA titers above the cut-off and GMTs (ACWY-TT and Men-PS groups)

Antibody	Estimate	Timing	ACWY-TT		Men-PS	
			*N*	Value (95 % CI)	*N*	Value (95 % CI)
MenA	% ≥1:8	Pre	30	6.7 (0.8, 22.1)	19	0 (0.0, 17.6)
		Month 1	30	100 (88.4, 100)	19	100 (82.4, 100)
		Year 1	30	93.3 (77.9, 99.2)	19	84.2 (60.4, 96.6)
		Year 2	99	94.9 (88.6; 98.3)	98	91.8 (84.5; 96.4)
		Year 4	312	86.5 (82.2; 90.1)	107	73.8 (64.4; 81.9)
		Year 5	299	**90.0 (86.0, 93.1)**	105	**74.3 (64.8, 82.3)**
	% ≥1:128	Pre	30	6.7 (0.8, 22.1)	19	0 (0.0, 17.6)
		Month 1	30	100 (88.4, 100)	19	100 (82.4, 100)
		Year 1	30	90.0 (73.5, 97.9)	19	78.9 (54.4, 93.9)
		Year 2	99	92.9 (86.0; 97.1)	98	87.8 (79.6; 93.5)
		Year 4	312	78.5 (73.5; 83.0)	107	61.7 (51.8; 70.9)
		Year 5	299	**86.3 (81.9, 90.0)**	105	**68.6 (58.8, 77.3)**
	GMT	Pre	30	2.7 (1.7, 4.3)	19	2.0 (2.0, 2.0)
		Month 1	30	4231.2 (2730.7, 6556.2)	19	1463.2 (886.5, 2415.0)
		Year 1	30	1066.9 (472.4, 2409.6)	19	218.0 (71.0, 669.6)
		Year 2	99	807.1 (559.5; 1164.2)	98	385.8 (259.4; 573.9)
		Year 4	312	278.6 (219.7; 353.2)	107	105.4 (67.6; 164.4)
		Year 5	299	**303.9 (248.2, 372.0)**	105	**103.6 (67.8, 158.3)**
MenC	% ≥1:8	Pre	30	10.0 (2.1, 26.5)	18	0.0 (0.0, 18.5)
		Month 1	30	100 (88.4, 100)	18	100 (81.5, 100)
		Year 1	30	96.7 (82.8, 99.9)	17	94.1 (71.3, 99.9)
		Year 2	100	98.0 (93.0; 99.8)	99	86.9 (78.6; 92.8)
		Year 4	312	88.5 (84.4; 91.8)	107	84.1 (75.8; 90.5)
		Year 5	299	79.3 (74.2, 83.7)	104	71.2 (61.4, 79.6)
	% ≥1:128	Pre	30	3.3 (0.1, 17.2)	18	0.0 (0.0, 18.5)
		Month 1	30	100 (88.4, 100)	18	100 (81.5, 100)
		Year 1	30	83.3 (65.3, 94.4)	17	94.1 (71.3, 99.9)
		Year 2	100	86.0 (77.6; 92.1)	99	79.8 (70.5; 87.2)
		Year 4	312	81.4 (76.6; 85.6)	107	73.8 (64.4; 81.9)
		Year 5	299	69.2 (63.7, 74.4)	104	65.4 (55.4, 74.4)
	GMT	Pre	30	3.1 (1.7, 5.6)	18	2.0 (2.0, 2.0)
		Month 1	30	6886.0 (4473.9, 10598.7)	18	8070.7 (4896.6, 13302.2)
		Year 1	30	462.7 (239.2, 895.2)	17	1956.8 (731.8, 5232.7)
		Year 2	100	304.4 (232.0; 399.5)	99	286.3 (181.8; 450.9)
		Year 4	312	273.6 (220.6; 339.4)	107	315.0 (196.8; 504.1)
		Year 5	299	114.0 (90.5, 143.5)	104	142.4 (85.3, 237.6)
MenW	% ≥1:8	Pre	30	3.3 (0.1, 17.2)	18	11.1 (1.4, 34.7)
		Month 1	30	96.7 (82.8, 99.9)	17	76.5 (50.1, 93.2)
		Year 1	30	93.3 (77.9, 99.2)	18	66.7 (41.0, 86.7)
		Year 2	100	84.0 (75.3; 90.6)	100	24.0 (16.0; 33.6)
		Year 4	312	74.0 (68.8; 78.8)	107	25.2 (17.3; 34.6)
		Year 5	299	**71.6 (66.1, 76.6)**	105	**24.8 (16.9, 34.1)**
	% ≥1:128	Pre	30	0.0 (0.0, 11.6)	18	5.6 (0.1, 27.3)
		Month 1	30	96.7 (82.8, 99.9)	17	76.5 (50.1, 93.2)
		Year 1	30	90.0 (73.5, 97.9)	18	61.1 (35.7, 82.7)
		Year 2	100	80.0 (70.8; 87.3)	100	19.0 (11.8; 28.1)
		Year 4	312	68.6 (63.1; 73.7)	107	20.6 (13.4; 29.5)
		Year 5	299	**64.9 (59.2, 70.3)**	105	**21.0 (13.6, 30.0)**
	GMT	Pre	30	2.1 (1.9, 2.5)	18	3.3 (1.6, 7.0)
		Month 1	30	9571.6 (4649.0, 19706.4)	17	881.6 (150.8, 5154.0)
		Year 1	30	1659.2 (728.5, 3778.7)	18	120.3 (23.6, 614.5)
		Year 2	100	257.8 (161.8; 410.7)	100	6.5 (4.2; 10.0)
		Year 4	312	175.1 (131.5; 233)	107	11.3 (7.8; 16.3)
		Year 5	299	**170.2 (124.7, 232.4)**	105	**11.7 (7.9, 17.1)**
MenY	% ≥1:8	Pre	28	28.6 (13.2, 48.7)	11	9.1 (0.2, 41.3)
		Month 1	27	100 (87.2, 100)	12	100 (73.5, 100)
		Year 1	28	96.4 (81.7, 99.9)	12	50.0 (21.1, 78.9)
		Year 2	100	86.0 (77.6; 92.1)	100	44.0 (34.1; 54.3)
		Year 4	309	82.8 (78.2; 86.9)	107	43.9 (34.3; 53.9)
		Year 5	299	**84.3 (79.7, 88.2)**	105	**44.8 (35.0, 54.8)**
	% ≥1:128	Pre	28	17.9 (6.1, 36.9)	11	9.1 (0.2, 41.3)
		Month 1	27	100 (87.2, 100)	12	100 (73.5, 100)
		Year 1	28	89.3 (71.8, 97.7)	12	33.3 (9.9, 65.1)
		Year 2	100	82.0 (73.1; 89.0)	100	36.0 (26.6; 46.2)
		Year 4	309	78.6 (73.6; 83.1)	107	34.6 (25.6; 44.4)
		Year 5	299	**80.9 (76.0, 85.2)**	105	**41.9 (32.3, 51.9)**
	GMT	Pre	28	9.3 (3.5, 24.5)	11	3.4 (1.2, 9.7)
		Month 1	27	3659.5 (2193.4, 6105.6)	12	2663.0 (1821.9, 3892.4)
		Year 1	28	1157.7 (572.2, 2342.3)	12	22.3 (3.4, 146.2)
		Year 2	100	367.1 (232.2; 580.2)	100	19.4 (11.4; 33.0)
		Year 4	309	350.5 (268.9; 456.7)	107	26.0 (16.6; 40.7)
		Year 5	299	**306.0 (236.3, 396.3)**	105	**29.6 (18.7, 46.7)**

Percentage of participants with rSBA antibody titers ≥1:8 and ≥1:128 and geometric mean titers in the ACWY-TT and the Men-PS groups at pre-vaccination, Month 1 and Year 1 (subset of participants from the ATP cohort for persistence Year 2 with samples tested by rSBA at the PHE laboratory) and at Years 2 (ATP cohort for persistence Year 2), 4 (ATP cohort for persistence Year 4) and 5 (Total Cohort Year 5)

ACWY-TT = group of participants who received one dose of MenACWY-TT at Month 0

Men-PS = group of participants who received one dose of MenACWY-PS at Month 0

ATP = according to protocol

Pre = pre-vaccination; Month 1 = 1 month post-vaccination, Year 1, 2, 4 and 5 = 1, 2, 4 and 5 years post-vaccination

*N* = number of participants with available results

% = percentage of participants with titers within the specified range

GMT = geometric mean titer

95 % CI = 95 % confidence interval

Bold: 95 % CI on group difference or GMT ratio excluding equality between the ACWY-TT and Men-PS groups

The results from the subset of samples retested at the PHE laboratory (collected at pre-vaccination, Year 1 and Year 2) suggested that the largest decrease in rSBA GMTs occurred during the first year following vaccination in both groups for all four serogroups (Table 2). Between Month 1 and Year 5, the rSBA GMTs decreased between 12.0- and 60.4-fold in the ACWY-TT group and between 14.1- to 90.0-fold in the Men-PS group. All rSBA GMTs at Year 5 were still above the pre-vaccination levels determined in the subset of samples retested at the PHE laboratory.

Exploratory analysis at Year 5 suggested that the percentages of participants with rSBA titers ≥1:8 and ≥1:128 and the rSBA GMTs for serogroups A, W and Y were significantly higher in the ACWY-TT group than in the Men-PS group (Table 2).

The data for each of the age strata were consistent with the data for the total population: in the ACWY-TT group, the percentage of adolescents (11–17 years of age) with rSBA titers ≥1:8 was 92.8, 80.3, 74.0, and 81.3 % for serogroups A, C, W and Y, respectively, while the percentage of adults (18–55 years of age) with rSBA titers ≥1:8 was 83.5, 76.9, 65.9, and 91.2 % for these serogroups, respectively; in the Men-PS group, the percentage of adolescents with rSBA titers ≥1:8 was 80.3, 65.8, 23.7, and 42.1 % for serogroups A, C, W and Y, respectively, while the percentage of adults with rSBA titers ≥1:8 was 58.6, 85.7, 27.6, and 51.7 % for serogroups A, C, W and Y, respectively (Additional file 1: Table S2).

### Safety

No SAEs related to vaccination or events related to the lack of efficacy were reported between Month 0 and Year 5.

Twenty-three study participants reported pregnancies between Year 3 and Year 5; one participant was diagnosed with eclampsia. All pregnancies had a normal outcome and resulted in birth of live infants with no apparent congenital anomalies.

## Discussion

This extension study reports the persistence of the immune response induced by a single dose of MenACWY-TT or a licensed MenACWY-PS in healthy adolescents and adults 5 years after vaccination. The majority of participants vaccinated with MenACWY-TT retained rSBA titers ≥1:8 against all four serogroups (71.6 % [serogroup W] to 90.0 % [serogroup A]) at Year 5 after vaccination, indicating that the protection continues for up to 5 years. This was observed for both the adolescent (11–17 years of age) and the adult (18–55 years of age) strata. The percentages of participants retaining seroprotective levels were observed to be lower in the MenACWY-PS recipients (ranging from 24.8 % [serogroup W] to 74.3 % [serogroup A]), with evidence for potential differences in seroprotective levels for serotypes A, C, W, and Y shown in an exploratory analysis.

Our persistence results are consistent with previously published data, which showed that at 3.5 years following a single dose of MenACWY-TT administered to adolescents (15–19 years old), all participants had rSBA titers ≥1:8 against all four serogroups [31]. Another study with a single dose of MenACWY-CRM in adolescents showed similar results at 5 years after primary vaccination; in the rSBA assay performed at PHE, 78 to 98 % of the MenACWY-CRM recipients retained rSBA titers ≥1:8 against serogroups A, W and Y at Year 5, and only 56 % of the participants against serogroup C [44].

Although IMD incidence is highest in infants, there is a secondary disease peak in adolescents [51]. In addition, the adolescent population plays an important role in the epidemiology of meningococcal disease as the main carrier group. Asymptomatic carriage is most common in adolescents and young adults [52–54], and since most transmission occurs through asymptomatic carriers, the adolescent population is an important reservoir for transmission. It was previously demonstrated that meningococcal serogroup C conjugate vaccines reduce asymptomatic carriage [17], therefore a vaccine that protects a high percentage of adolescents may lead to lower incidence of IMD in other age-groups.

Exploratory analyses suggested that the percentage of participants with rSBA titers ≥1:8 or ≥1:128, and the rSBA GMTs were significantly higher in the ACWY-TT group than in the Men-PS group for serogroups A, W and Y at 5 years after vaccination. For serogroup C, the results were comparable in terms of percentages of participants with seroprotective levels and rSBA GMTs in both groups, and there was no evidence of a significant difference. This is in line with the results reported in the primary study for the Year 3 timepoint [30] and with a previous study which also showed that the rSBA GMTs of MenACWY-TT were significantly higher than those of MenACWY-PS for serogroups A, W, and Y in participants aged 18–55 years [34]. Several previous studies with all three quadrivalent conjugate vaccines (MenACWY-TT, MenACWY-DT and MenACWY-CRM) reported evidence for higher antibody titers in the recipients of conjugate vaccines as compared to those induced by polysaccharide vaccines [22, 27, 44, 55, 56]. While several of these studies evaluated GMTs after a short period following vaccination, there is evidence that higher GMTs established initially correspond to higher proportions of participants retaining the protective bactericidal antibody threshold during long-term follow-up [42]. This observation is valid for the current study as well, especially for serogroup W, where a low initial GMT value in the MenPS group correlated with a low percentage of participants (~25 %) who retained seroprotective rSBA-MenW titers at Year 5 post-vaccination. The waning of antibody titers between 1 and 2 years post-vaccination with MenACWY-PS suggests that an earlier (2 years) than currently recommended (5 years) revaccination may be warranted in individuals who remain at high risk of exposure to *N. meningitidis*, preferably with a meningococcal conjugate vaccine.

During this study, no SAEs related to vaccination were reported and all pregnancies had normal outcomes, suggesting that the long-term safety and reactogenicity profile of MenACWY-TT was clinically acceptable. No AEs due to lack of vaccine efficacy were reported, but this needs to be interpreted in the light of the fact that no meningococcal invasive disease outbreaks have occurred in the Philippines or in Saudi Arabia since 2006 [38].

The present study was limited by its open design, which could have introduced bias in the safety reporting; however, since no safety concerns were raised in either group, this was probably not a major drawback. The study was also potentially limited by the exploratory nature of the statistical comparisons, which should be interpreted with caution, considering that the study was not designed for inferential comparisons at Year 5, there was no adjustment for multiplicity of comparisons, and the clinical relevance of these differences is unknown. Antibody titers were measured using an rSBA assay, due to the practical advantages of a commercially available heterologous complement source. However, it is known that the use of rabbit complement as opposed to human complement results in a higher absolute value of the titers [42, 44, 48]. Finally, only a limited number of samples from pre-vaccination, Month 1, Years 1 and 2 were retested at PHE. Although a direct comparison should be made cautiously because of inter-laboratory procedural differences, different sample sizes, and a different number of freeze-thaw cycles for a given clinical sample, the re-analysis at PHE gave lower point estimates compared to the original rSBA assay performed at GSK Vaccines [30].

## Conclusions

This study showed that MenACWY-TT, when administered as a single intramuscular dose to adolescents and adults 11–55 years of age, induced an immune response against meningococcal serogroups A, C, W and Y, which persisted in the majority of participants up to 5 years after vaccination regardless of age stratum. The long-term immunogenicity for serogroups A, W and Y was observed to be higher in the conjugated vaccine group compared to the polysaccharide vaccine group.

*Menactra* is a trademark of Sanofi Pasteur.

*Nimenrix*, *Menveo*, and *Mencevax* are trademarks of the GSK Group of companies.

## Additional file

Additional file 1: Table S1. Percentage of participants with rSBA antibody titers above the cut-off and GMTs (ATP cohort for persistence Year 5). **Table S2**. Percentage of participants per age strata with rSBA titers above the cut-off and GMTs (Total Cohort Year 5). Percentage of participants per age strata with rSBA antibody titers ≥1:8 and ≥1:128 and geometric mean titers in the ACWY-TT and the Men-PS groups at pre-vaccination, at Years 4 and 5 (Total Cohort Year 5). (DOC 111 kb)

## Abbreviations

ATP: According-to-protocol
CI: Confidence interval
CRM_197_: *Corynebacterium diphtheriae* toxin
DT: Diphtheria toxoid
FDA: Food and Drug Administration
GMT: Geometric mean antibody titer
MenACWY: Quadrivalent meningococcal vaccine
MenPS: Meningococcal polysaccharide
PHE: Public Health England
rSBA: Rabbit complement
SAE: Serious adverse event
TT: Tetanus toxoid
