# Supplementary material for: Long-term immunogenicity and safety after a single dose of the quadrivalent meningococcal serogroups A, C, W, and Y tetanus toxoid conjugate vaccine in adolescents and adults: 5-year follow-up of an open, randomized trial
Source: BMC Infect Dis. 2015 Oct 6;15:409. doi: 10.1186/s12879-015-1138-y (PMC4595195; doi:10.1186/s12879-015-1138-y)
Supplement: Additional file 1: Table S1. — Percentage of participants with rSBA antibody titers above the cut-off and GMTs (ATP cohort for persistence Year 5). Table S2. Percentage of participants per age strata with rSBA titers above the cut-off and GMTs (Total Cohort Year 5). Percentage of participants per age strata with rSBA antibody titers ≥1:8 and ≥1:128 and geometric mean titers in the ACWY-TT and the Men-PS groups at pre-vaccination, at Years 4 and 5 (Total Cohort Year 5). (DOC 111 kb) [file 12879_2015_1138_MOESM1_ESM.doc]

**Table S1.** Percentage of participants with rSBA antibody titers above the cut-off and GMTs (ATP cohort for persistence Year 5)

| **Antibody** | **Estimate** | **Timing** | **ACWY-TT** | | **Men-PS** | |
| --- | --- | --- | --- | --- | --- | --- |
|  |  |  | **N** | **Value (95% CI)** | **N** | **Value (95% CI)** |
| **MenA** | % ≥1:8 | Year 4 | 43 | 95.3 (84.2–99.4) | 17 | 76.5 (50.1–93.2) |
|  |  | Year 5 | 51 | 84.3 (71.4–93.0) | 19 | 57.9 (33.5–79.7) |
|  | % ≥1:128 | Year 4 | 43 | 86.0 (72.1–94.7) | 17 | 58.8 (32.9–81.6) |
|  |  | Year 5 | 51 | 76.5 (62.5–87.2) | 19 | 47.4 (24.4–71.1) |
|  | GMT | Year 4 | 43 | 365.0 (225.8–589.9) | 17 | 104.4 (31.0–351.3) |
|  |  | Year 5 | 51 | 189.8 (107.7–334.6) | 19 | 37.0 (12.6–108.7) |
| **MenC** | % ≥1:8 | Year 4 | 43 | 76.7 (61.4–88.2) | 17 | 41.2 (18.4–67.1) |
|  |  | Year 5 | 51 | 72.5 (58.3–84.1) | 18 | 38.9 (17.3–64.3) |
|  | % ≥1:128 | Year 4 | 43 | 62.8 (46.7–77.0) | 17 | 23.5 (6.8–49.9) |
|  |  | Year 5 | 51 | 54.9 (40.3–68.9) | 18 | 27.8 (9.7–53.5) |
|  | GMT | Year 4 | 43 | 126.0 (61.6–257.7) | 17 | 16.7 (5.7–48.7) |
|  |  | Year 5 | 51 | 78.5 (41.8–147.4) | 18 | 17.3 (6.0–49.7) |
| **MenW** | % ≥1:8 | Year 4 | 43 | 90.7 (77.9–97.4) | 17 | 17.6 (3.8–43.4) |
|  |  | Year 5 | 51 | 86.3 (73.7–94.3) | 19 | 31.6 (12.6–56.6) |
|  | % ≥1:128 | Year 4 | 43 | 72.1 (56.3–84.7) | 17 | 17.6 (3.8–43.4) |
|  |  | Year 5 | 51 | 74.5 (60.4–85.7) | 19 | 31.6 (12.6–56.6) |
|  | GMT | Year 4 | 43 | 240.0 (128.1–449.6) | 17 | 8.3 (3.6–19.5) |
|  |  | Year 5 | 51 | 281.6 (145.9–543.2) | 19 | 15.4 (5.7–41.9) |
| **MenY** | % ≥1:8 | Year 4 | 43 | 86.0 (72.1–94.7) | 17 | 47.1 (23.0–72.2) |
|  |  | Year 5 | 51 | 92.2 (81.1–97.8) | 19 | 63.2 (38.4–83.7) |
|  | % ≥1:128 | Year 4 | 43 | 86.0 (72.1–94.7) | 17 | 47.1 (23.0–72.2) |
|  |  | Year 5 | 51 | 92.2 (81.1–97.8) | 19 | 57.9 (33.5–79.7) |
|  | GMT | Year 4 | 43 | 442.9 (229.8–853.4) | 17 | 30.7 (9.0–105.2) |
|  |  | Year 5 | 51 | 769.7 (438.6–1351.0) | 19 | 74.1 (21.9–250.3) |

Footnote: ACWY-TT = group of participants who received one dose of MenACWY-TT at Month 0

Men-PS = group of participants who received one dose of MenACWY-PS at Month 0

ATP = according to protocol

Years 4 and 5 = 4 and 5 years post-vaccination

N = number of participants with available results

%= percentage of participants with titers within the specified range

GMT = geometric mean titer

95% CI = 95% confidence interval

**Table S2. Percentage of participants per age strata with rSBA titers above the cut-off and GMTs (Total Cohort Year 5)**

Percentage of participants per age strata with rSBA antibody titers ≥1:8 and ≥1:128 and geometric mean titers in the ACWY-TT and the Men-PS groups at pre-vaccination, at Years 4 and 5 (Total Cohort Year 5)

|  |  |  | **11–17 years age stratum** | | | | **18–55 years age stratum** | | | |
| --- | --- | --- | --- | --- | --- | --- | --- | --- | --- | --- |
| **Antibody** | **Estimate** | **Timing** | **ACWY-TT** | | **Men-PS** | | **ACWY-TT** | | **Men-PS** | |
|  |  |  | **N** | **Value (95% CI)** | **N** | **Value (95% CI)** | **N** | **Value (95% CI)** | **N** | **Value (95% CI)** |
| **MenA** | % ≥1:8 | Year 4 | 204 | 86.8 (81.3, 91.1) | 75 | 76.0 (64.7, 85.1) | 81 | 86.4 (77.0, 93.0) | 28 | 67.9 (47.6, 84.1) |
|  |  | Year 5 | 208 | **92.8 (88.4, 95.9)** | 76 | **80.3 (69.5, 88.5**) | 91 | **83.5 (74.3, 90.5)** | 29 | **58.6 (38.9, 76.5)** |
|  | % ≥1:128 | Year 4 | 204 | 78.4 (72.1, 83.9) | 75 | 62.7 (50.7, 73.6) | 81 | 79.0 (68.5, 87.3) | 28 | 64.3 (44.1, 81.4) |
|  |  | Year 5 | 208 | **90.4 (85.5, 94.0)** | 76 | **73.7 (62.3, 83.1)** | 91 | **76.9 (66.9, 85.1)** | 29 | **55.2 (35.7, 73.6)** |
|  | GMT | Year 4 | 204 | 288.3 (213.4, 389.5) | 75 | 111.4 (65.3, 190.1) | 81 | 243.2 (155.9, 379.4) | 28 | 99.9 (39.1, 255.2) |
|  |  | Year 5 | 208 | **394.8 (316.2, 493.0)** | 76 | **131.6 (81.9, 211.4)** | 91 | **167.1 (110.9, 251.8)** | 29 | **55.4 (22.3, 137.6)** |
| **MenC** | % ≥1:8 | Year 4 | 204 | 88.7 (83.6, 92.7) | 75 | 82.7 (72.2, 90.4) | 82 | 89.0 (80.2, 94.9) | 28 | 85.7 (67.3, 96.0) |
|  |  | Year 5 | 208 | **80.3 (74.2, 85.5)** | 76 | **65.8 (54.0, 76.3)** | 91 | 76.9 (66.9, 85.1) | 28 | 85.7 (67.3, 96.0) |
|  | % ≥1:128 | Year 4 | 204 | 81.4 (75.3, 86.5) | 75 | 70.7 (59.0, 80.6) | 82 | 84.1 (74.4, 91.3) | 28 | 82.1 (63.1, 93.9) |
|  |  | Year 5 | 208 | 68.3 (61.5, 74.5) | 76 | 57.9 (46.0, 69.1) | 91 | 71.4 (61.0, 80.4) | 28 | 85.7 (67.3, 96.0) |
|  | GMT | Year 4 | 204 | 268.5 (207.9, 346.7) | 75 | 235.6 (133.8, 414.9) | 82 | 356.0 (226.2, 560.3) | 28 | 706.4 (274.1, 1820.4) |
|  |  | Year 5 | 208 | 111.3 (84.9, 145.9) | 76 | 90.5 (49.7, 164.8) | 91 | 120.4 (77.3, 187.7) | 28 | 487.3 (201.4, 1179.1) |
| **MenW** | % ≥1:8 | Year 4 | 204 | 76.5 (70.0, 82.1) | 75 | 20.0 (11.6, 30.8) | 82 | 70.7 (59.6, 80.3) | 28 | 35.7 (18.6, 55.9) |
|  |  | Year 5 | 208 | **74.0 (67.5, 79.9)** | 76 | **23.7 (14.7, 34.8)** | 91 | **65.9 (55.3, 75.5)** | 29 | **27.6 (12.7, 47.2)** |
|  | % ≥1:128 | Year 4 | 204 | 72.1 (65.4, 78.1) | 75 | 17.3 (9.6, 27.8) | 82 | 62.2 (50.8, 72.7) | 28 | 28.6 (13.2, 48.7) |
|  |  | Year 5 | 208 | **70.2 (63.5, 76.3)** | 76 | **18.4 (10.5, 29.0)** | 91 | **52.7 (42.0, 63.3)** | 29 | **27.6 (12.7, 47.2)** |
|  | GMT | Year 4 | 204 | 218.2 (152.9, 311.4) | 75 | 9.2 (6.1, 13.8) | 82 | 114.7 (66.5, 197.9) | 28 | 17.7 (7.3, 42.5) |
|  |  | Year 5 | 208 | **230.1 (159.1, 332.9)** | 76 | **10.7 (6.9, 16.6)** | 91 | **85.5 (48.7, 150.0)** | 29 | **14.5 (6.3, 33.3)** |
| **MenY** | % ≥1:8 | Year 4 | 203 | 83.3 (77.4, 88.1) | 75 | 45.3 (33.8, 57.3) | 80 | 83.8 (73.8, 91.1) | 28 | 42.9 (24.5, 62.8) |
|  |  | Year 5 | 208 | **81.3 (75.3, 86.3)** | 76 | **42.1 (30.9, 54.0)** | 91 | **91.2 (83.4, 96.1)** | 29 | **51.7 (32.5, 70.6)** |
|  | % ≥1:128 | Year 4 | 203 | 77.8 (71.5, 83.3) | 75 | 34.7 (24.0, 46.5) | 80 | 82.5 (72.4, 90.1) | 28 | 35.7 (18.6, 55.9) |
|  |  | Year 5 | 208 | **77.4 (71.1, 82.9)** | 76 | **39.5 (28.4, 51.4)** | 91 | **89.0 (80.7, 94.6)** | 29 | **48.3 (29.4, 67.5)** |
|  | GMT | Year 4 | 203 | 337.6 (243.2, 468.6) | 75 | 24.9 (15.0, 41.5) | 80 | 461.4 (275.6, 772.6) | 28 | 30.5 (11.1, 83.5) |
|  |  | Year 5 | 208 | **231.6 (168.6, 318.2)** | 76 | **25.2 (15.0, 42.5)** | 91 | **578.4 (379.9, 880.6)** | 29 | **44.7 (16.8, 118.9)** |

Footnote: ACWY-TT = group of participants who received one dose of MenACWY-TT at Month 0

Men-PS = group of participants who received one dose of MenACWY-PS at Month 0

Year 4 and 5= 4 and 5 years post-vaccination

N = number of participants with available results

%= percentage of participants with titers within the specified range

GMT = geometric mean titer

95% CI = 95% confidence interval

**Bold:** 95% CI on group difference or GMT ratio excluding equality between the ACWY-TT and Men-PS groups
